# Evaluation of different intramuscular injectable anesthetic combinations in rabbits: Impact on anesthetic depth, physiological parameters, and EEG recordings

**DOI:** 10.1371/journal.pone.0319106

**Published:** 2025-02-25

**Authors:** Marie-Louise Schmid, Julia Werner, Anna M. Saller, Judith Reiser, Yury Zablotski, Julian Ostertag, Matthias Kreuzer, Christine Lendl, Heidrun Potschka, Christine Baumgartner

**Affiliations:** 1 Center of Preclinical Research, TUM School of Medicine and Health, Technical University of Munich, Munich, Bavaria, Germany; 2 Clinic for Swine, Center for Clinical Veterinary Medicine, Ludwig-Maximilians-Universität München, Oberschleißheim, Bavaria, Germany; 3 Department of Anesthesiology & Intensive Care, TUM School of Medicine and Health, Technical University of Munich, Munich, Bavaria, Germany; 4 Tiergesundheitszentrum München, Munich, Bavaria, Germany; 5 Institute of Pharmacology, Toxicology, and Pharmacy, Ludwig-Maximilians-Universität München, Munich, Bavaria, Germany; 6 Veterinary Faculty, Ludwig-Maximilians-Universität München, Munich, Germany; Research Institute for Brain and Blood Vessels, Akita Cerebrospinal and Cardiovascular Center, JAPAN

## Abstract

Rabbits have a high anesthesia-related mortality rate because of their small size, high metabolic rate and challenging airway management. This study aimed to investigate different combinations of intramuscularly administered anesthetics in New Zealand White rabbits, focusing on their effects on anesthetic depth, physiological parameters, and electroencephalogram (EEG) recordings. Defined doses ketamine (K), esketamine (SK), medetomidine (M), dexmedetomidine (D), midazolam (Mi), and butorphanol (B) were investigated and compared in five different combinations: KM (25/0.25 mg/kg), SKM (17/0.25 mg/kg), SKD (17/0.15 mg/kg), MMiB (0.25/1/0.2 mg/kg), and DMiB (0.15/1/0.2 mg/kg). For 60 minutes, the anesthetic depth was assessed using an anesthesia score, and physiological parameters, including heart rate (HR), respiratory rate (RR), oxygen saturation and blood pressure were recorded. The study also assessed the latencies to loss and recovery of reflexes after targeted antagonization, and EEGs were measured. The rabbits were not intubated and were supplied with oxygenated air via nasal probes. All anesthetic combinations achieved anesthesia with surgical tolerance, with significant intergroup differences in HR, RR, blood pressure and EEG power spectra. The KM group demonstrated the most stable anesthesia and rapid recovery, while SKD and SKM groups showed prolonged recovery times. Oxygen saturation remained consistently high across all groups, obviating the need for intubation. All rabbits recovered fully after anesthesia. In conclusion, intramuscular administered anesthetic combinations can provide effective anesthesia with surgical tolerance for short procedures in rabbits. Monitoring circulatory parameters during and after anesthesia and adequate pain management pre-, inter-, and postoperatively are essential. Considering these criteria, the KM group presented the best results compared with the other groups.

## 1. Introduction

Rabbits are the third most frequently anesthetized animals in veterinary medicine after dogs and cats [1]. However, general anesthesia in rabbits is associated with high morbidity and mortality. Anesthesia-related mortality is many times higher in rabbits (0.73%) than in dogs (0.05%) and cats (0.11%) [2]. Most deaths during rabbit anesthesia are not attributable to a specific cause, but cardiorespiratory events are cited as the leading cause in approximately 40% of these cases [2]. Their small body size predisposes rabbits to hypothermia and bradycardia, while their high metabolic rate, their high susceptibility to stress and difficult airway management adds further challenges [3–6]. A comparatively small heart in relation to body size is an additional risk factor of anesthesia-related death in rabbits [6]. With increasing body weight, a corresponding decrease in heart rate indicates an inverse correlation between heart rate and body weight [7]. Given the necessity of sedation or anesthesia for numerous interventions, veterinarians must be able to plan and administer appropriate anesthesia for rabbits to ensure the safety and well-being of the animal during the procedure [8]. Rabbits are obligate nose breathers [9]. However, the induction of anesthesia with inhalation anesthetics via a face mask is stressful for rabbits [4]. Moreover, it can lead to irregular breathing associated with bradycardia, hypercapnia, and hypoxemia [4,10]. These breathing irregularities can also occur when propofol is used as the sole anesthetic for intravenous anesthesia. Therefore, its use as a long-term anesthetic is not recommended [4,11]. Procedures necessitating anesthesia typically require an unobstructed airway [12]. However, intubation in rabbits can be challenging [12]. Stress and airway management difficulties compound the risks as rabbits prominent, owing to their large incisors, long and narrow oral cavity, thick tongue, and limited mobility of the temporomandibular joint, the larynx is difficult to visualize, and securing the airway is difficult [13]. Non-invasive airway management, with oxygen supplementation via nasal probes offers a viable alternative to mitigate these issues [14]. This study aimed to investigate an institutional anesthetic combination of SK and M, as current knowledge is mainly based on personal experience and a single study describing its nasal application [15]. This earlier study compared it with a KM combination, and further studies reported favorable outcomes under KM anesthesia [16–18].

The primary objective was to assess anesthesia quality under spontaneous respiration, as previous studies focused on intubated animals [15–17]. The institutional combination was compared to other regimens within a standardized setup.

To establish comparative anesthetic combinations, other similar studies were referenced. One found a depressive effect of the DK combination on blood pressure and HR compared to its use with Mi [19]. Another study used MMi and fentanyl, achieving surgical tolerance in 14 out of 19 animals [16]. Due to fentanyl’s controlled substance status, it was avoided in this study, which explored combining Mi with B as an alternative and considered replacing M with its more potent analogon D [20]. Furthermore, all anesthetics were administered intramuscularly in this study, whereas other studies have employed varying routes of administration [15–18,21,22]. This study aimed to investigate the quality and side effects of five intramuscular injectable anesthetic combinations in achieving surgical tolerance while ensuring physiological stability and rapid recovery under spontaneous breathing conditions.

## 2. Materials and methods

The study complied with the EU Directive 2010/63/EU for animal experiments and the German Animal Welfare Act (2018) [23]. The Ethical Committee for Animal Experiments of the Government of Upper Bavaria, Munich, Germany approved the animal experiments. To minimize animal use, the rabbits were subsequently included in other projects (Reference Numbers ROB-55.2-2532.Vet_02-19-139, ROB-55.2-2532.Vet_02-20-219, ROB-55.2-2532.Vet_02-21-51, ROB-55.2-2532.Vet_02-21-181).

### Animals

Thirty-five non-castrated male New Zealand White rabbits with a mean body weight of 3.28 kg ±  0.35 kg standard deviation (SD) and an age of 5 to 6 months were purchased from a commercial supplier (Charles River, France). The rabbits were housed individually under conventional hygienic conditions in cages (Tecniplast R-SUITE X-type, Tecniplast Deutschland GmbH, Hohenpeißenberg, Germany) with perforated Noryl shelves (WxDxH: 653 ×  653 ×  95 mm) and a platform (WxDxH: 70 ×  30 ×  27 cm) providing a second level for resting on top or hiding underneath. In addition, the rabbits were offered a regular free range in the enclosure. The room temperature ranged between 18 and 21 °C, and the humidity ranged between 45 and 65%. A 12-hour light and 12-hour night cycle was used to simulate the natural daily rhythm. Pellet feed (Altromin Spezialfutter GmbH & Co. KG, Lage, Germany), hay and hay cobs, and tap water were available ad libitum. For enrichment, a wooden chew stick (SAFE® block gnawing, J. Rettenmaier & Söhne GmbH +  Co KG, Rosenberg, Germany) was provided as an enrichment agent in the home cage. The rabbits were single-housed and had olfactory, visual, and auditory contact with their fellow rabbits.

During the study, a score sheet was used to assess the achievement of humane endpoints. For this purpose, the animals were examined on two consecutive days after anesthesia. Particular attention was paid to BCS, food and water intake, circulatory parameters and the injection site for anesthesia. In our study, no animal reached the humane endpoint, so no animal was euthanized. All animals left the study in good health.

### Preparation for anesthesia

The animals were subjected to a clinical examination, which included measuring body weight. The fur on both ears was locally shaved at the vascular access area, and anesthetic cream (Emla®, lidocaine 25 mg/g cream, prilocaine 25 mg/g cream, AstraZeneca GmbH, Wedel, Germany) was applied locally 30 minutes before the start of the experiment. The preparations involved inserting catheters (Vasofix® Safety 20 G, 33 mm, B. Braun Melsungen AG, Melsungen, Germany) into the central auricular ear artery of the right ear and the lateral auricular vein of the left ear (Vasofix® Safety 22 G, 25 mm, B. Braun Melsungen AG, Melsungen, Germany).

### Study design

The study was designed and conducted as a pilot study. Accordingly, the number of animals was set to n =  7, as previous experiments demonstrated that this sample size provided sufficiently valid data. This decision was made in accordance with regional regulatory authorities, who were involved in the approval process. The persons conducting the study were blinded to the data analysis. Simple randomization was performed with a computer program.

### Drug treatment and methods

The rabbits were randomly assigned to five experimental groups with seven animals each. In the KM group, rabbits received K 25 mg/kg (Ketamin 100 mg/ml cp Pharma, Burgdorf, Germany) and M 0.25 mg/kg (Sedator 1 mg/ml Dechra, Aulendorf, Germany). The SKM group was treated with SK 17 mg/kg (Ketanest S 25 mg/ml Pfizer, Berlin, Germany) and M 0.5 mg/kg; the SKD group received SK 17 mg/kg and D 0.15 mg/kg (Dexmopet 0.5 mg/ml alfavet, Neumünster, Germany). The MMiB group received M 0.25 mg/kg, Mi 1 mg/kg (Midazolam HEXAL 5 mg/ml, Hexal, Holzkirchen, Germany) and B 0.2 mg/kg (Butorgesic 10 mg/ml cp Pharma, Burgdorf, Germany), and the DMiB group received D 0.15 mg/kg, Mi 1 mg/kg and B 0.2 mg/kg. The anesthetic drug combinations were drawn up in a syringe, and the solubility of the substances was checked macroscopically and then injected into the left quadriceps femoris muscle. Injection volumes of more than two ml were split equally between both hind limbs. After the administration of the respective anesthetic combination, the rabbit was placed in a cage in the dark with a conventional warming mat. For this pilot study, each rabbit was anesthetized only once; afterward, they were used in other studies.

### Measurements

The cage was equipped with a camera (AC420 Action Camera 1080P Wi-Fi Full HD, Govicture, Guangdong, China) to record the induction and recovery phases. After loss of the righting reflex (LOR), the rabbits were placed on an operating table equipped with a warming mat in a thoracoabdominal position. Eye ointment (Vitamycin® Augensalbe, CP-Pharma, Burgdorf, Germany) was applied, and the rabbits were preoxygenated at an oxygen rate of 1 liter/min. A pulse oximeter (2500A VET, Nonin Medical Inc., Minnesota, USA) was placed on the toe of the right hind limb. Heparin 150 I.E./kg (Heparin-Sodium 5.000 I.E./ml, B. Braun Melsungen AG, Melsungen, Germany) and metamizole 65 mg/kg (Metamizol WDT 500 mg/ml, WDT, DE, Germany) were administered intravenously. The rabbits received an infusion (Ringer Infusionslösung B. Braun Melsungen AG, Melsungen, Germany) with a flow rate of 10 ml/kg/h. For the EEG recordings, two recording electrodes (Single Subdermal Needle Electrode, 27 G, 13 mm, Friendship Europe ApS, Roskilde, Denmark) were positioned subcutaneously in a shaved area 1 cm caudal to the lateral canthus of the eye, and a reference electrode was placed on the midline of the frontal bone [24]. EEGs were recorded continuously at a sampling rate of 128 Hz (Narcotrend®-Compact M, Narcotrend-Gruppe, Hannover, Germany). During the entire recording period, the impedances were less than 2 kΩ. After the EEG electrodes were positioned, the rabbits were placed in a supine position. On the basis of the description by Henke et al. [14], venous catheters were modified as nasal probes to measure the RR and expiratory CO2 concentration. For this purpose, a venous catheter without a mandrain (Vasofix® Safety 18 G, 45 mm, B. Braun Melsungen AG, Melsungen, Germany) was inserted into the left nostril and connected to a capnograph (Datex-Ohmeda S/5, GE Healthcare, Munich, Germany) (S1). A second venous catheter without a mandrain (Vasofix® Safety 22 G, 25 mm, B. Braun Melsungen AG, Melsungen, Germany), was inserted into the right nostril as a nasal probe for the administration of oxygen (0.5 liter/min) (S1). To measure blood pressure, the arterial ear catheter was connected to an invasive blood pressure monitoring kit (Druckmess-Set, 1-fach, CODAN pvb Medical GmbH, Forstinning, Germany), a calibrated blood pressure sensor APT300 and a PLUGSYS TAM-A transducer amplifier module (Hugo Sachs Elektronik - Harvard Apparatus GmbH, March-Hugstetten, Germany) at heart level. An electrocardiogram (ECG) with needle electrodes (ECG/EMG cable with exchangeable needles for ECGA (Rodents) Monopolar Subdermal needle 0.35 mm in diameter and 30 mm in length, Hugo Sachs Elektronik – Harvard Apparatus GmbH, March-Hugstetten, Germany) was attached subcutaneously at the right and left thorax as well as at the left abdomen. A temperature probe (Flexible Vinyl Rectal Thermocouple Probe for Rabbits and Large Animals, Hugo Sachs Elektronik – Harvard Apparatus GmbH, March-Hugstetten, Germany) was inserted rectally to record body temperature (T). Systolic (SAP), diastolic (DAP) and mean arterial pressure (MAP), HR, and T were consistently monitored every four seconds using compatible hardware modules and their respective software (PLUGSYS, heart rate module, Thermocouple Amplifier Module, HAEMODYN Software v2.0, Hugo Sachs Elektronik – Harvard Apparatus GmbH, March-Hugstetten, Germany). Starting 10 minutes after the induction of anesthesia, defined parameters were investigated every 5 minutes to evaluate the depth of anesthesia via a specific scoring system (Table 1).

**Table 1:** Scoring system for assessing the depth of anesthesia.

Parameter	Examination Method		points
Whisker Movement (WM)	Whiskers were brushed against their direction of growth with a cotton swab, and their movement was observed.	present moderate low absent	0 1 2 3
Bulbus rotation (BR)	Bulbus rotation toward the nasal eye corner was evaluated.	no low moderate high	0 1 2 3
Prolapse of the nictitating membrane (PM)	The visibility of the nictitating membrane was evaluated.	no low moderate high	0 1 2 3
Corneal reflex (CR)	The cornea was touched with a cotton swab, then the eyelid closure was evaluated.	present absent	0 1
Lid response (LR)	The medial eye corner was touched with a cotton swab; then, the eyelid closure was evaluated.	present absent	0 1
Muscle tone (MT)	A single flexion and extension of the hind limb determined muscle tension.	high reduced relaxed	0 1 2
Ear pinch reflex (EPR)	Pressure was applied to the base of the ear with the thumb and index finger for 3 seconds and the response was assessed.	present moderate absent	0 1 2
Withdrawal reflex hind limb (WRH)	An analgesia meter (Rodent Pincher - Analgesia Meter, Bioseb, FR, France) applied pressure between the toes for 3 seconds and the response was assessed.	present moderate absent	0 1 2
Withdrawal reflex front limb (WRF)		present moderate absent	0 1 2
Maximum Score Points			19

A maximum score of 19 points could be achieved at each time point, with 0 points representing a fully conscious rabbit.

### Recovery phase

After 60 min, the KM, SKM, and SKD groups were antagonized with 1.25 mg/kg atipamezole (Antisedan 5 mg/ml, Vetoquinol GmbH, Ismaning, Germany). The MMiB and DMiB groups received 1.25 mg/kg atipamezole and 0.1 mg/kg flumazenil (Flumazenil-hameln, hameln pharma GmbH, Hameln, Germany). The right quadriceps femoris muscle was chosen as the site of application. Injection volumes of more than two ml were split equally between both hind limbs. After antagonization, the rabbits continued to be scored, and scores were taken every five minutes until they reached zero. The monitoring devices were then removed, and the rabbit was returned to the video-monitored cage. The time it took to regain the reflexes (ROR) after antagonization and to regain the ability to stand and walk (ASW) were recorded. As soon as the rabbits maintained sufficient oxygen saturation without oxygen supplementation, they were returned to their home cages. The body weight was measured the following day, two days, and one week after anesthesia.

### Data processing and statistical analysis

Using HAEMODYN software, measurements, including SAP, DAP, MAP, HR, and T, were recorded every four seconds and, after the end of the experiments, exported to Excel (Microsoft® Excel® für Microsoft 365 MSO Version 2403, Build 17425.20236). The mean MAP and HR values were calculated over one-minute periods beginning 30 seconds after the reflex check (WRF) to assess hemodynamics during anesthesia. RR was monitored via the Datex-Ohmeda S/5 and documented manually. Anesthesia scores were determined by summing the points from the individual parameters and were calculated every five minutes over 11 time points (tp) until antagonization. After antagonization, only the anesthesia score was further assessed. Considering the repeated measures, generalized linear mixed effects models with individual animals as a random effect were chosen to analyze the MAP, HR, RR, and anesthesia score. The following model assumptions were always checked: (1) the Shapiro test was used to check the normality of the residuals-Wilk normality test, (2) the homogeneity of variances between groups was checked with the Bartlett test, and (3) heteroscedasticity (constancy of error variance) was checked with the Breusch–Pagan test. If the assumption was satisfied, generalized linear mixed-effects models were used (R package - Elmer). The R package Elmer computes weighted estimates via the design adaptive scale and thus solves heteroskedastic and nonnormally distributed residuals by assigning lower weights to outliers and other variables that skew the data. Additionally, both linear and robust linear models were compared using six main performance quality indicators: Akaike’s information criterion (AIC), the Bayesian information criterion (BIC), the conditional coefficient of determination R2, the marginal coefficient of determination R2, the intraclass-correlation coefficient (ICC) and the root mean square error (RMSE). The model showing the best combination of predictive (AIC and BIC) and fitting (explanatory, R2, ICC, and RMSE) power was preferred. All contrasts (differences) between particular tps (e.g., minute 10 or 55) and between particular treatments (e.g., KM or SKD) were assessed after model fitting by the estimated marginal means (R package - emmeans) with Tukey p value correction for multiple comparisons. The results with a p value <  0.05 were considered statistically significant. Data analysis was performed using R software version 4.3.3 (2024-02-29). The figures were created with GraphPad Prism 10.3.0 (507).

The normal distributions of LOR, ROR, ASW, weight, T, oxygen saturation, and pressure of pinching were tested via the Shapiro‒Wilk normality test. Normally distributed data were analyzed with an ordinary one-way ANOVA. A Kruskal-Wallis test was carried out for nonparametric data. Data was analyzed and graphs were created using GraphPad Prism 10.3.0 (507).

EEG data were processed using MATLAB R2023a (MathWorks, Inc., Natick, MA, United States). The raw EEG data were filtered from 0.5 to 47 Hz via a Butterworth forward‒backward zero‒phase bandpass routine with the MATLAB *filtfilt* function. Artifacts and EEG burst suppression patterns were removed from the filtered EEG data. Two artifact-free minutes were selected halfway through the anesthesia period for further analysis. The power spectral density of the selected EEG episodes was calculated using the MATLAB *pwelch* function. The MATLAB-based MES toolbox [25] was used to calculate the area under the receiver operating characteristic curve (AUC) as an effect size to compare PSD at different tps. AUC values range from 0 to 1 and are interpreted with respect to their difference from 0.5. AUC values can be roughly interpreted as excellent: 1 ≥  AUC ≥  0.9; good: 0.9>  AUC ≥  0.8; fair: 0.8>  AUC ≥  0.7; poor: 0.7>  AUC ≥  0.6; or fail: AUC < 0.6 [26]. Values less than 0.5 can be converted to this scale by subtracting the AUC value from 1. To determine statistical relevance, AUC values were 10000-fold bootstrapped, and 95% confidence intervals for the AUC values were determined. 5. Statistically significant differences were assumed if the AUC 95% confidence interval was less than or equal to 0.5. Significant results were additionally defined by at least two neighboring frequencies showing relevant differences in the comparison of power spectral density. To mitigate the risk of obtaining false positive results due to multiple comparisons, previously described procedures were applied [27–29].

## 3. Results

The mean body weights ±  SDs of the rabbits were: KM, 3.37 ±  0.22 kg; SKM, 3.03 ±  0.27 kg; SKD, 3.24 ±  0.33 kg; MMiB, 3.01 ±  0.13 kg; and DMiB, 3.20 ±  0.30 kg, with no significant differences between groups (Table 2).

**Table 2:** Body weights of the rabbits over time.

Day	KM	SKM	SKD	MMiB	DMiB
0	3.37 ± 0.22	3.03 ± 0.27	3.24 ± 0.33	3.01 ± 0.13	3.20 ± 0.30
1	3.30 ± 0.21	3.00 ± 0.26	3.19 ± 0.31	2.98 ± 0.14	3.18 ± 0.31
2	3.32 ± 0.22	3.00 ± 0.27	3.20 ± 0.33	2.96 ± 0.13	3.17 ± 0.28
7 ± 3	3.39 ± 0.26	3.12 ± 0.21	3.50 ± 0.56	3.06 ± 0.12	3.30 ± 0.29

Mean weight in kg ±  SD on experimental day 0, days 1 and 2, and one week later ±  3 days.

For a rabbit weighing 3.28 kg anesthesia injection volumes were: MMiB, 1.5 ml; KM, 1.6 ml; DMiB, 1.7 ml; SKM, 3.1 and SKD, 3.2. Antagonization volumes averaged: KM, SKM and SKD, 0.8 ml; MMiB and DMiB, 4.1 ml (Table 3).

**Table 3:** Injection volumes for rabbits with an average weight of 3.28 kg.

Group	Anesthesia	Antagonization	Average injection volume	
	volume per kg	volume per kg	Anesthesia	Antagonization
KM	0.5 ml	0.25 ml	1.6 ml	0.8 ml
SKM	0.9 ml	0.25 ml	3.1 ml	0.8 ml
SKD	1.0 ml	0.25 ml	3.2 ml	0.8 ml
MMiB	0.5 ml	1.25 ml	1.5 ml	4.1 ml
DMiB	0.5 ml	1.25 ml	1.7 ml	4.1 ml

### Course and depth of anesthesia

**Fig 1:**
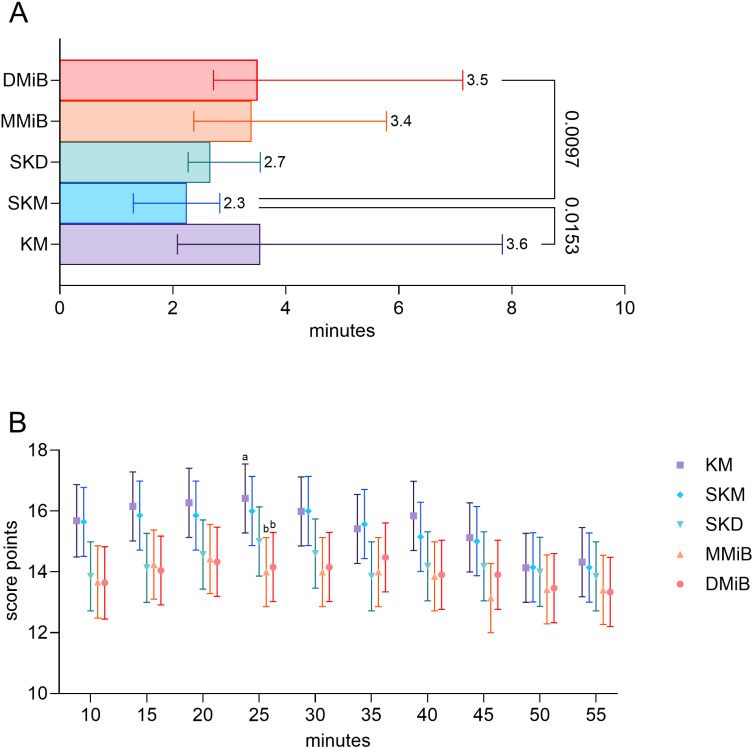
Loss of the righting reflex and anesthesia scores. A. Time from anesthesia induction to LOR, presented as medians ±  95% confidence intervals. Statistical analysis was performed using the Kruskal-Wallis test and Dunn’s multiple comparisons test for group-wise differences (p <  0.05). B. Anesthesia scores from 10 to 55 minutes post induction, analyzed using robust linear mixed models. Group comparisons were conducted after model fitting, with estimated marginal means and Tukey’s p value correction for multiple comparisons. Significant differences (p <  0.05) are indicated by superscripts within a time point. n =  7 per group.

The shortest latency to loss of the righting reflex was evident in the SKM group (2.3 ±  0.63 min) (Fig 1A).

The longest latencies to LOR were observed in the KM (3.60 ±  2.00 min, p =  0.0153) and DMiB (3.50 ±  1.80 min, p =  0.0097) groups, with intermediate latencies in the MMiB and SKD groups (3.40 ±  1.19 min and 2.70 ±  0.47 min), respectively.

Anesthesia was maintained for 60 minutes in all the groups, with absent responses to EPR, WRF, and WRH, only reappearing after antagonization. Pinching pressure for WRF (949.50 ±  170.60 g) and WRH (990.50 ±  169.50 g) did not differ significantly among the groups at the individual tps.

The KM and SKM groups had the highest anesthesia scores overall with significant differences at minute 25, where KM scored higher than MMiB (p =  0.0263) and DMiB (p =  0.0467). Reflex sensitivity ratings for limbs, ears and muscle tone were typically 2 while eye reflexes varied, with an LR and CR score of 1 for most animals and scores of 2-3 in the WM. All the animals regained consciousness and completely recovered after antagonization.

### Physiological parameters

**Fig 2:**
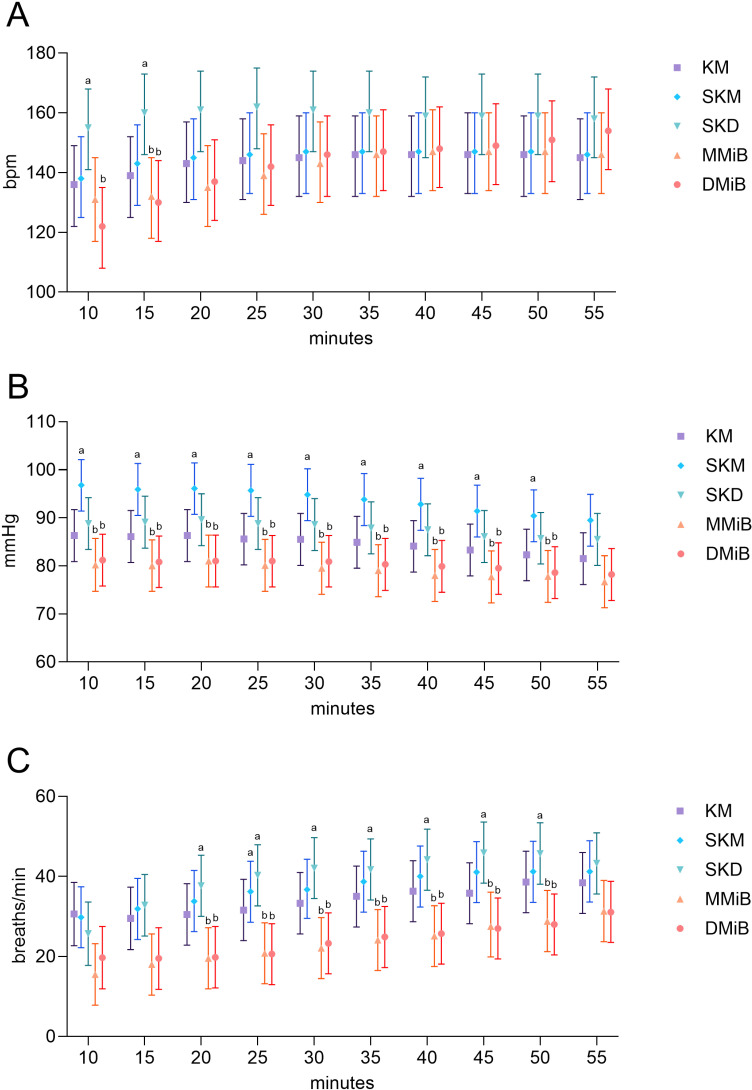
Physiological parameters over time. A. Mean HR (bpm), B. MAP (mmHg) and C. Mean RR (breaths per minute) with estimated means ±  95% confidence intervals. Robust linear mixed models were used for analysis, with Tukey’s p-value correction for multiple comparisons. Superscripts indicate significant differences (p <  0.05) between groups. The p-values for MAP and RR are provided in the supplements (S2, S3 Tables). n =  7 per group.

Significant differences in HR among the groups were observed at 10 and 15 min (Fig. 2A). SKD rabbits showed higher HRs than DMiB rabbits at 10 min (p =  0.0058) and both DMiB (p =  0.022) and MMiB (p =  0.031) rabbits at 15 min (Fig 2A). Sinus arrhythmias occurred in two animals (MMiB, DMiB) within the first 10 min and in one animal (MMiB) during the whole procedure. After 50 min, one animal (SKM) presented slight sinus arrhythmias, and another animal (DMiB) presented single supraventricular extrasystoles.

In all the experimental groups, the RR increased over time (Fig 2C). Shortness of breath, observed in three rabbits (2 in SKD, 1 in DMiB) during induction of anesthesia, was treated via mechanical stimulation of the breathing (swiveling around the transverse axis and pinching the nasal septum).

Oxygen saturation remained stable (99%, SD: 94-100%), with no significant group differences.

### EEG recordings

**Fig 3:**
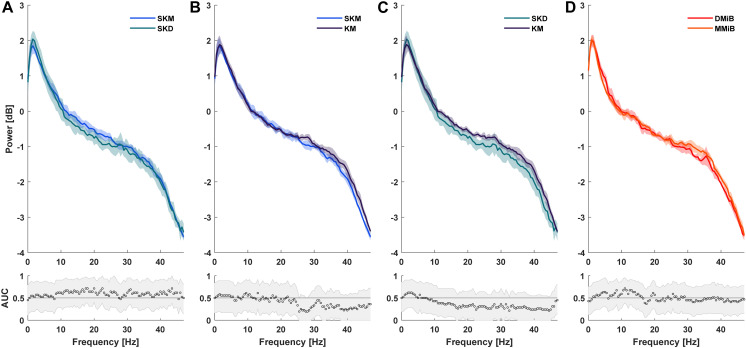
Group comparisons of EGG recordings. Group comparisons of EGG recordings were analyzed 30 minutes after anesthesia induction over a 2- minute interval. Statistically significant differences were assumed if the AUC 95% confidence interval was ≤  0.5.

All groups exhibited a characteristic EEG pattern with high power at lower frequencies and low power at higher frequencies. No significant differences were found in power spectra among K/SK groups (SKM, SKD, KM) (Figs 3A–3C) or between nonketamine groups (MMiB and DMiB) (Fig 3D).

**Fig 4:**
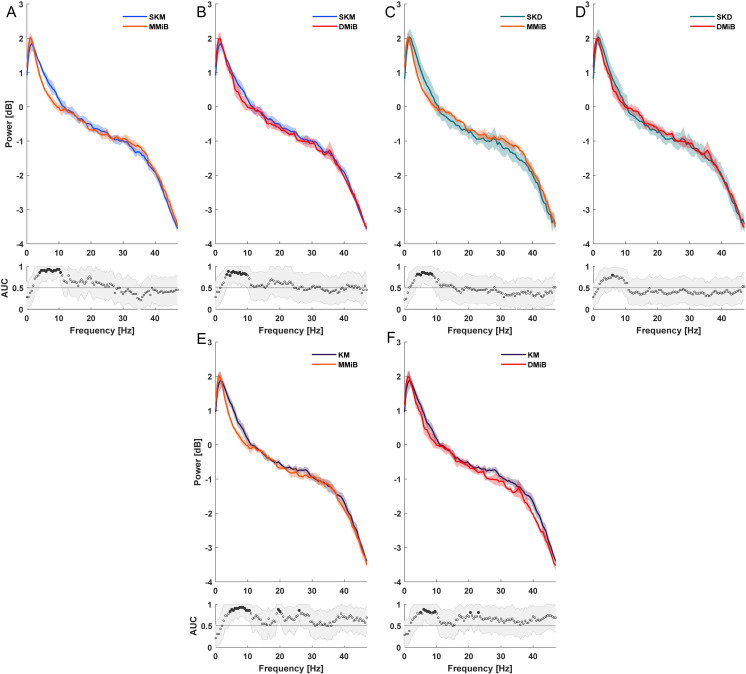
Spectrograms and AUC comparison. Spectrograms and AUCs analyzed 30 minutes post induction for 2 minutes, significant differences were assumed if the AUC 95% confidence interval was ≤  0.5.

Significant differences in the power spectrum were noticeable between K/SK groups (SKM, SKD, and KM) and nonketamine groups (MMiB, DMiB). K/SK groups (SKM, KM) presented significantly increased power at ~ 3-10 Hz compared to MMiB and DMiB (Figs 4A, 4B, 4E, 4F). The SKD group differed significantly only from MMiB, not from DMiB (Figs 4C,4D).

### Recovery phase

**Fig 5:**
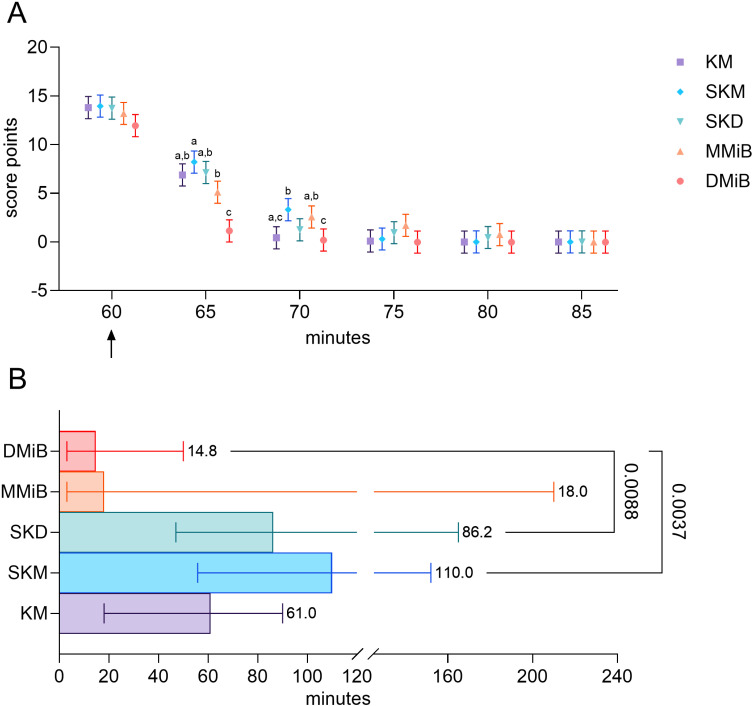
Recovery phase. A. Anesthesia scores were evaluated every five minutes after antagonization (↑) until all reflexes were regained. Robust linear mixed models analyzed group differences, with Tukey’s p-value correction applied. Superscripts indicate significant differences (p <  0.05) between groups. B. Time (in minutes) until ASW recovery, analyzed using a Kruskal-Wallis test with Dunn’s multiple comparisons test for median contrasts. Values are medians ±  95% confidence intervals (n =  7 per group).

**Fig 6:**
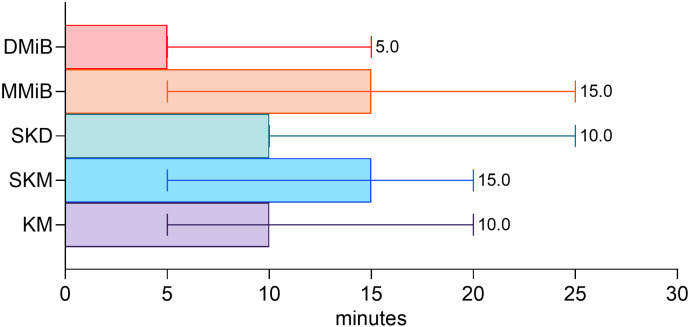
Regain of Reflexes. Time until full reflex recovery varied significantly among groups.

The DMiB group showed the fastest recovery, with significantly lower anesthesia scores within 5 min after antagonization compared to all groups (DMiB-KM p < .0001, DMiB-MMiB p < .0001, DMiB-SKD p < .0001, DMiB-SKM p < .0001, MMiB-SKM p =  0.0015) (Fig 5A). Reflex recovery times were the fastest for DMiB (5 min), followed by the KM and SKD (10 min each) and, MMiB and SKM (15 min each) (Fig 6).

DMiB group regained ASW fastest with a median time of 14.80 min, followed by MMiB (18.00 min), KM (61.00 min), and SKD (82.20 min). SKM exhibited the longest recovery (median 110 minutes) (Fig 5B). All animals recovered completely, though some required repeated antagonization (2 in MMiB, 1 in SKD, and 1 in SKM). One MMiB rabbit remained drowsy until the next day, despite prompt reflex response and normal physiological parameters. Various laboratory tests being conducted but the reason could not be identified. No significant difference in weight gain or loss were observed among the groups on days 1, 2, or 7 post anesthesia (Table 2). One DMiB rabbit vocalized during antagonization, and two MMiB rabbits twitched.

## 4. Discussion

### Course and depth of anesthesia

Unconsciousness, muscle relaxation, and analgesia are key elements of general anesthesia [30], requiring a combination of drugs. As in previous studies, we defined surgical tolerance as absent EPR, WRH and WRF [16,17]. LOR occurred later with Mi and B than with K or SK, consistent with prior findings [21], but the clinical relevance of these minor time differences remains uncertain. Additional reflexes (BR, CR, PM, LR) were recorded per Guedel classification [31]. While LR and CR are typically present during surgical tolerance [31], their absence in our study, suggest excessively deep anesthesia. Differences in anesthetic scores between KM, SKM compared to SKD may be attributable to the lower D dosage. Although D is described as twice as potent as M [32], there is no description of the effect on the parameters recorded in the score when used at half dose in combination with other drugs.

In contrast to previous studies [18,19], WRF reappeared first and before the other withdrawal reflexes during recovery. This is probably due to its prolonged persistence during induction [33] and reversed anesthesia stages during awakening [34]. Thus, the WRF appeared to be the most sensitive indicator of anesthetic depth in our study.

Although intramuscular injection is advantageous, as drugs administered via this route are absorbed rapidly, it is painful for the animals [35]. Depending on the injection frequency and volume, tissue damage in the muscle increases proportionally [36]. Therefore, a maximum injection volume of 1 ml per injection site for intramuscular injections in rabbits is recommended [37]. Currently, SK is only available in Germany at a concentration of 25 mg/ml, resulting in high injection volumes for the induction of anesthesia (Table 3). The high antagonization volume of 4.1 ml on average must also be considered for the DMiB and MMiB combinations (Table 3). In our study we divided the antagonization between two injection sites in favor of fewer injections. What remains to be discussed is whether the higher volume or two further injections represent a greater burden for the animal. To avoid intramuscular application, antagonists could also be administered subcutaneously [38,39] or intravenously [40] where higher volumes (2.5 ml/kg (subcutaneous), 2 ml/kg (intravenous)) [37] can be administered.

### Physiological parameters

Stable anesthesia with minimal physiological changes is essential for safe anesthesia [4]. HR and MAP remained within physiological ranges across all groups [38,41,42]. However, in MMiB and DMiB groups HR, MAP, and RR were consistently lower, with RR falling below the physiological range. Respiratory depression associated with B in rabbits has been reported in several studies [18,43]. Schroeder and Smith noted reduced RR with Mi, B or their combination [44]. When Mi was used with low doses of D, it caused minimally respiratory impairment and induced sedation [45,46]. A flattening of oxygen saturation was also observed with intranasal administration of MiB or DB [47]. Transnasal administration of DMiB resulted in 45 minutes of surgical anesthesia but impaired breathing, with a 70% deviation from baseline [48], likely due to the administration route. In our study, the maintenance of SpO2 within physiological limits across all groups underscores the adequacy of oxygen supplementation via nasal probes as recommended by Henke et al. [14]. This is particularly important for injection anesthesia if intubation is not possible due to the procedure [14]. The findings suggest that nasal oxygen supplementation is sufficient to prevent hypoxemia, even in groups with reduced RR (MMiB, DMiB). Blood pressure was higher during anesthesia with SK and M compared in combination with K, likely due to the pharmacology of SK, an enantiomer of the racemic anesthetic K [49]. The s (+)-isomer has a fourfold greater binding affinity to the NMDA receptor, resulting in fewer side effects and stronger analgesic effects [49,50]. This increased binding affinity [49] could enhance the sympathomimetic effect of K [39], potentially resulting in increased blood pressure. This effect may provide hemodynamic stability during anesthesia but necessitates careful monitoring in animals predisposed to hypertension. The higher MAP could also explain the improved perfusion observed in tissues, potentially contributing to better overall anesthetic depth [50]. In contrast, the MMiB and DMiB groups demonstrated lower MAP levels, likely due to the combined depressant effects of Mi and B on vascular tone [44,48]. While these combinations are effective for premedication sedation [33] and managing painful procedures, their use requires caution in patients with underlying cardiovascular or respiratory conditions [44,48].

### Adverse events

M can cause hypotension, bradycardia, and cardiac arrhythmias in many species, especially after intravenous administration [51]. We also observed arrhythmias in individual animals of each group, except the KM group, after intramuscular application. However, cardiac events had no impact on blood pressure or the stability of anesthesia.

Respiratory side effects of the drugs used in this study are well described [22,44,52]. However, the respiratory events that occurred in the SKD group during induction may not be drug related. Rabbits are nose breathers, and their nostrils contain sensory pads at the entrance, making the nose very sensitive to touch [9]. Inserting the probes for capnometry and oxygen supply could therefore have provoked brief respiratory arrest, especially as this mostly occurred during the insertion of the catheters at the beginning of anesthesia.

### EEG recordings

The spectrogram can be segmented into delta (0-4 Hz), theta (4–8 Hz), alpha (8–12 Hz), beta (12–30 Hz), and gamma bands ( > 30 Hz) [53]. The inverse relationship between frequency and power observed here aligns with the 1/f shape found in human EEG [54]. Given similarities between rodent and human EEGs [55] the recorded measurements likely reflect physiologic patterns. Higher delta activity was noted in SKD, SKM, and KM groups compared to MMiB and DMiB, consistent with states of sleep or unconsciousness [56]. Studies in rats show that K increases delta power, shortly after administration [57], and similar effect with SK have been observed [58]. Our findings suggest K and SK induce similar EEG changes in rabbits. Conversely, M had no effect on oscillatory activity in the delta range during inhalation anesthesia with isoflurane [59]. This finding is consistent with reduced delta power in the MMiB and DMiB groups. Opioids like alfentanil increase EEG activity, particularly in the delta range [60], but it remains unclear if butorphanol has similar effects in rabbits. For now, the observed differences are attributed to SK and K.

### Recovery phase

Given their susceptibility to complications during the recovery phase, a brief recovery period from anesthesia is crucial for rabbits owing to their unique anatomy and physiology [1]. However, we observed that the MMiB and DMiB groups appeared to wake up quite abruptly from deep anesthesia, which we perceived as stressful for the animals. Reducing the recovery phase lowers the risk of hypothermia [6], while minimizing stress in rabbits prevents catecholamine-induced sympathotonus, which can impair intestinal motility [4,39] and hemodynamics [38]. SK has a faster metabolism and shorter recovery phase than K [61], so faster recovery was expected in the SKD and SKM groups compared to KM. However, our results could not confirm this finding. A possible explanation is the delayed antagonization of alpha-2 agonists, which occurred only after 60 minutes. If SK was used, antagonization could have been done earlier (30–45 minutes) [38]. The delay, due to the standardized protocol, allowed M and D an additional 15–30 minutes to exert their circulatory depressant effects without SK [51].

### Limitations

Since we investigated only healthy male animals of the same age, we cannot draw conclusions about the stability of anesthesia and side effects in female animals, different age groups, or those with preexisting conditions. In addition, we used a small number of animals in this pilot study.

## 5. Conclusions

Intramuscular combination anesthesia is feasible in healthy male New Zealand White rabbits under spontaneous respiration with nasal oxygen supply for short procedures requiring surgical tolerance. Oxygen saturation remained stable across all combinations, and nasal probe insertion caused no clinical changes. The WRF was the most sensitive indicator of surgical tolerance. KM proved the optimal combination, leading to stable circulatory parameters, rapid onset and a calm but rapid recovery, with no respiratory or cardiac side effects. In MMiB and DMiB groups, the RR fell below physiological limits, but oxygen saturation remained stable due to continuous oxygen supply. SKM and SKD groups experienced prolonged recovery phases. EEG spectrogram revealed greater delta activity in K/SK groups compared to B and Mi groups, indicating that K and SK affect the EEG pattern in rabbits, consistent with rat studies. Close monitoring of breathing and circulatory parameters is essential. Intubation is not mandatory but is recommended. Careful observation of the recovery and appropriate analgesic management pre-, inter- and postoperatively are essential.

## Supporting information

S1 Fig Schematic illustration of the nasal probes (created with BioRender.com). (TIFF)

S2 Table Significant differences in MAP. (PDF)

S3 Table Significant differences in RR. (PDF)

S4 Table RawValues_vital signs_anesthetic score_weight. (XLSX)

S5 Table RawValues_ASW_LOR_ROR_pinching pressure. (XLSX)

S6 Table RawValues_EEG. (XLSX)
