# Supplementary figures and images for: Evaluation of different intramuscular injectable anesthetic combinations in rabbits: Impact on anesthetic depth, physiological parameters, and EEG recordings
Source: PLoS One. 2025 Feb 25;20(2):e0319106. doi: 10.1371/journal.pone.0319106 (PMC11856588; doi:10.1371/journal.pone.0319106)

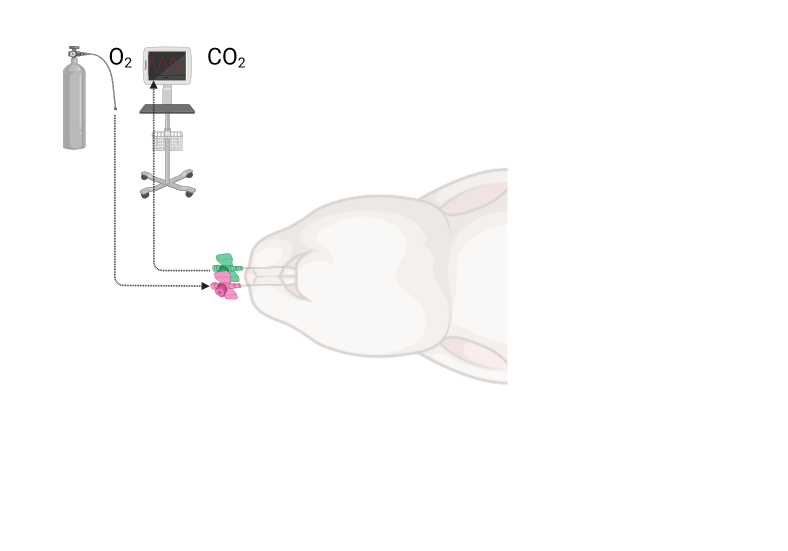

Supplement: S1 Fig — (TIFF) [file pone.0319106.s001.tiff]
