# Supplementary material for: Evaluation of different intramuscular injectable anesthetic combinations in rabbits: Impact on anesthetic depth, physiological parameters, and EEG recordings
Source: PLoS One. 2025 Feb 25;20(2):e0319106. doi: 10.1371/journal.pone.0319106 (PMC11856588; doi:10.1371/journal.pone.0319106)
Supplement: S2 Table — (PDF) [file pone.0319106.s002.pdf]

Table S2: Significant differences in MAP.

**MAP p.value**	group	
time	SKM-MMiB	SKM-DMiB
10	0.0002	0.0006
15	0.0004	0.0010
20	0.0010	0.0010
25	0.0006	0.0014
30	0.0008	0.0034
35	0.0014	0.0047
40	0.0014	0.0081
45	0.0039	0.0179
50	0.0105	0.0203
55	0.0085	0.0298

Group comparisons SKM-MMiB and SKM DMiB show the p.values at the individual time points. Values belong to Figure 2B.
