# Supplementary material for: Evaluation of different intramuscular injectable anesthetic combinations in rabbits: Impact on anesthetic depth, physiological parameters, and EEG recordings
Source: PLoS One. 2025 Feb 25;20(2):e0319106. doi: 10.1371/journal.pone.0319106 (PMC11856588; doi:10.1371/journal.pone.0319106)
Supplement: S3 Table — (PDF) [file pone.0319106.s003.pdf]

Table S3: Significant differences in RR.

**RR p.value**	group			
time	SKD-MMiB	SKD-DMiB	SKM-DMiB	SKM-MMiB
10				
15				
20	0.0088	0.0109		
25	0.0037	0.0032	0.0366	0.0417
30	0.0026	0.0057		
35	0.0119	0.0185		
40	0.0049	0.0072		
45	0.0072	0.0052		
50	0.0189	0.0117		

Group comparisons of SKD-MMiB, SKD-DMiB, SKM-DMiB and SKM-MMiB show the p.values at the individual time points. Values belong to Figure 2C.
